# Large Language Models in Psychiatry: A Systematic Review of Clinical Use Cases and Evidence

**DOI:** 10.1192/bjo.2026.11093

**Published:** 2026-06-30

**Authors:** James McLeod, Cen Cong, Mennatullah Dakroury, Sadhana Prabhu, Judith Harrison

**Affiliations:** 1 Newcastle University, Newcastle upon Tyne, United Kingdom; 2 NHS, Newcastle upon Tyne, United Kingdom

## Abstract

**Aims:**

Large language models (LLMs) are rapidly emerging as tools with potential applications across mental healthcare, yet their implications for psychiatric practice remain unclear. This systematic review explores and categorises LLM use cases in mental healthcare to inform clinicians, researchers, and policymakers about emerging applications, opportunities, evidence gaps, and safety challenges relevant to psychiatric assessment, risk management, clinical documentation, and patient-facing support.

**Methods:**

We searched EMBASE, MEDLINE, PsycINFO, PubMed, the ACL Anthology, the ACM Digital Library, arXiv, medRxiv, and bioRxiv (2017–June 2025) and included empirical studies evaluating LLMs for mental healthcare tasks. Screening and extraction wereperformed in line with PRISMA. We summarised use cases using a predefined taxonomy and recorded study design, evaluation setting (synthetic vs clinical), and key safety/ethics issues.

**Results:**

We identified 120 studies, with some addressing more than one use case, reflecting a broad and rapidly expanding set of LLM applications in mental health care. The most common functions related to assessment and detection: symptom identification (14), early detection (15), risk stratification (e.g. suicidality) (9), individual-level risk prediction for developing mental health disorders (12), and analysis of clinical or patient-generated text (1). Patient-facing use cases included psychotherapy (10), conversational agents (15), patient education (6), mood and emotion monitoring (5), accessibility-focused digital interventions (2), and psychiatric rehabilitation (1). Clinician-facing functions included diagnostic support (18), disease classification (7), treatment outcome prediction (3), electronic health record summarisation (3), and documentation support (1). Additional work addressed healthcare policy (8), research (1), education (1), and training (1). Across domains, LLMs were frequently reported to perform comparably to existing automated approaches and, in standardised case evaluations, to human benchmarks. However, the evidence base was highly variable, most studies were exploratory or proof-of-concept and had limited evaluation in routine psychiatric practice. Recurrent concerns included hallucinated or inaccurate outputs, bias and cultural insensitivity, limited transparency and explainability, data privacy risks, and uncertain safety in high-risk clinical contexts.

**Conclusion:**

Current evidence indicates that LLMs may support multiple areas in psychiatry, primarily as augmentative tools rather than replacements for clinical expertise. Promising near-term applications include assessment support, administrative efficiency, psychoeducation, and low-risk supportive interactions. However, clinical integration remainsat an early stage given limited real-world validation and persistent safety concerns. Future research should prioritise evaluation in routine care, strengthened governance and regulatory frameworks, and transparent systems co-designed with clinicians and service users to ensure safe and equitable implementation in psychiatric practice.

